# Acute Inversion of the Uterus Following Delivery

**Published:** 1945

**Authors:** Coralie Rendle-Short

**Affiliations:** Tutor in Obstetrics, University of Bristol; Senior Obstetrical Officer, Southmead Hospital


### ACUTE INVERSION OF THE UTERUS FOLLOWING DELIVERY.

BY

CORALIE RENDLE-SHORT, M.B., Ch.M., M.R.C.O.G.

*Tutor in Obstetrics, University of Bristol;*
*Senior Obstetrical Officer, Southmead Hospital.*

---

ACUTE inversion of the uterus following delivery is one of the most rare complications of labour. According to Eden and Holland it only occurs in 1 in 180,000 to 1 in 200,000 labours. The following three cases have been dealt with in recent years at Southmead Hospital, Bristol; curiously enough the first two both took place within the same week, in the odd way which rarities have of occurring in pairs.

*Case* 1. Mrs. O. P., aged 29.

She had had one previous pregnancy, two and a half years ago, which was normal except for a severe post-partum hæmorrhage with a partially adherent placenta that necessitated manual removal.

During the present pregnancy she was attended by her own doctor in a country district. Labour commenced normally at 2 p.m. on 6th September, 1940, and he was called in at 8 p.m. to find the head on the perineum and the contractions strong. He immediately delivered her of a healthy male infant weighing 7 lb.; there was a second degree tear of the perineum. He waited twenty minutes and then without handling the fundus told the patient to strain down. She did so, and to his surprise the whole uterus came down outside the vulva, completely inverted, with the placenta attached. Hastily, without any "scrubbing up" he detached the placenta and pushed back the uterus, as he thought, to its normal position. The patient by that time was very shocked, and was treated by warmth and rectal saline. She improved somewhat, but there was a fair amount of bleeding throughout the night.

The following morning she was transferred sixteen miles to this Hospital by ambulance. On arrival at 11 a.m. she was found to be grey, cold and shocked, and was losing a fair amount of dark red blood per vaginum. The pulse was 112, of very poor volume, and her temperature was 98.4 deg. She was treated by warmth and morphia, and was given an immediate transfusion of two pints of Group "O" Blood; when she had recovered sufficiently an examination was made. Abdominally the uterus was just palpable 2 inches above the symphysis pubis; it was tender, flat at the top, and a "dent" could be made out. Vaginally there was some dark red bleeding. On internal examination a soft mass was felt protruding down into the vagina; the cervix could not be made out.

At 7 p.m. the patient was taken to the theatre and anæsthetized with gas and oxygen. She was put up in lithotomy position, and the vulva and surrounding parts painted with picric acid in spirit. The vagina was exposed and the swollen œdematous bleeding mass could be seen; this was the uterus completely inverted. Blood clots were removed, and the whole area painted with picric acid solution; then by gently squeezing the uterus to reduce the œdema, the uterus was slowly replaced, the fundus going back last. Once reduction had been started it was fairly simple. Ergometrine 1 c.cm., and a hot intra-uterine douche were given. The condition at the end of the operation was satisfactory. She was given 10 c.cm. of anti-streptococcal serum (concentrated globulin) intra-muscularly, at once, and this was repeated the next morning.

The patient then, contrary to expectations, made an uneventful recovery. In spite of the grave risk of sepsis she only developed a temperature of 99.4 deg. on the third morning, and that immediately fell to normal. The lochia was a little offensive for the first five days, but was normal in amount, and soon cleared up. The uterus was involuted below the symphysis pubis by the 8th day. She was given iron and vitamins, but no sulphonamides. She was discharged well on 22nd September, 1940.

*Case* 2. Mrs. P. P., aged 27.

She was a primigravida—37 weeks—admitted at 3 p.m. on 11th September, 1940, in labour. She had been attended at home by her own doctor, and had been in bed with severe pyelitis for one month. She had a history of having had a stone removed from the left kidney three years previously.

On admission the patient looked ill and pale, and was complaining of frequent "sweating attacks," and acute pain in the left kidney region. The temperature was 98.2 deg., pulse 128. The urine was scanty, loaded with albumin and contained blood; she had frequent rigors after admission to hospital. She was treated by massive doses of alkalies, fluids by mouth, and morphia when necessary.

Labour proceeded normally until 2 p.m. on 12th September, 1940, when the cervix was fully dilated, and owing to her poor condition an anæsthetic was given, and a normal female infant weighing 5 lb. 10 oz. was delivered by forceps.

Immediately following delivery there was severe post-partum hæmorrhage. The uterus could not be felt abdominally so a hand was inserted into the vagina, and a partially detached placenta was felt in the uterus. A manual removal was performed with some difficulty, as part of the placenta was very adherent.

Ergometrine was given, but the uterus would not contract well; there was hardly any bleeding. By 7 o'clock, four hours later, her condition was very unsatisfactory. There was a continuous trickle of dark blood from the vagina, and it was still not possible to feel the uterus per abdomen. A second general anæsthetic was given, and a vaginal examination made, and a completely inverted uterus was found. This was replaced and continuous intra-venous saline given, followed by a transfusion of two pints of blood.

She immediately started to improve, and eventually made a very good recovery. The temperature never rose above 100 deg., and the pulse soon settled. By 21st September, 1940, her hæmoglobin was 74 per cent. ; the uterus was involuting well. She was discharged in a satisfactory condition on 10th October, 1940.

*Case* 3. Mrs. D. C., aged 20, primipara.

This girl had a perfectly uneventful pregnancy, and was sent to the country annexe of this Hospital for delivery.

On 20th April, 1944 she went into labour, and at 1.15 a.m. on the 21st was delivered of a living male child weighing 6 lb., the first stage lasting 5¼ hours, and the second stage 1½ hours. There was not very much bleeding, and ten minutes later the midwife expelled the placenta. She stated that it appeared complete, but the membranes were retained, and tore away from the edges as she delivered it. Following this the patient was rather shocked and lost several large clots of blood ; ergometrine 1.c cm. was given, followed by liquid extract of ergot. She then collapsed completely, and the pulse was imperceptible, so medical aid was sought at 1.50 a.m., and anacardone given. Morphia ¼ gr. was ordered by 'phone, and given immediately.

On arrival at 2.30 a.m. I found the patient cold, pulseless, and a blue-grey colour. There were many dark clots on the sheet. It was not possible to feel the fundus of the uterus, but every time supra-pubic pressure was made more dark clots came from the vagina. The blood-pressure was 20/? A pint of blood was immediately run into a vein in the right leg, followed by a ½ pint of plasma. The blood-pressure rose to 75/?, and she became warmer, and the pulse could be felt at the wrist. It was still not possible to feel the uterus at all, and the unusual dark vaginal bleeding had increased to a steady trickle.

As she was somewhat improved it seemed advisable to explore the uterus. There were two possibilities, one that the uterus was inverted, or secondly, that a succenturiate placenta had been left behind. A general anæsthetic was given, a catheter passed, and a hand inserted into the vagina, with full sterile precautions. A soft mass could be felt, filling the vagina, which was recognized to be the inverted uterus. It was replaced with some difficulty, and having got it back it would not retract at all well, and it was not possible to feel it abdominally, in spite of the fact that she was a very thin woman. A hot intra-uterine douche of normal saline was given, and a further 1 c.cm. of ergometrine. The retained membranes were peeled off. At the end of the operation her condition was not good, and she was cold and clammy. A further pint of blood was given slowly, and by 5.10 a.m. she was very much better. The blood-pressure was 90/60, pulse 130, and there was practically no loss vaginally ; still the fundus of the uterus could not be felt.

The patient made a very good recovery ; she developed some pyrexia (100 deg.) on the 3rd and 4th days of the puerperium, and was given sulphonamides by mouth, 2 grams initially, and then 1 gram. 4 hourly for four days: also iron, vitamins and osteocalcium. The interesting thing was that it was not possible to feel the uterus definitely per abdomen until the 5th day of the puerperium.

A vaginal examination was made on the 16th day; the lochia was alba, and the uterus involuted below the symphysis pubis, anteverted and not tender. She was allowed home on the 18th day, with her baby, which was breast-fed 3 hourly. A second post-natal examination was made six weeks after delivery; she appeared very well and had no trouble at all. The uterus was small, anteverted, and, if anything, was rather super-involuted. The cervix was in perfect condition.

These three cases, particularly the last, well illustrate the condition of acute inversion of the uterus. Traditionally it is supposed to occur as a result of pulling on the cord, but that was certainly not the cause here; it is more likely that it was due to pressure on the soft fundus. But when one sees the pressure that is sometimes used in trying to expel a placenta by tough midwives or doctors, one would have thought the accident would be much more frequent. No doubt the uterus is dimpled by the fingers, and it then contracts and turns itself inside out. Books describe degrees of inversion, partial and complete; I have on several occasions felt a depression in the uterus on trying to express an adherent placenta, and have immediately massaged the uterus and made sure it was very hard before trying again. In such a case, no doubt, the uterus could easily have become completely inverted. The fact however that pulling on the cord may cause the uterus to turn inside out is often demonstrated at Cæsarean Section, when deliberately delivering the placenta by pulling on the cord under direct vision. The uterus can be seen to start inverting itself, usually at the fundus or on the posterior wall.

The diagnosis of the condition is not always easy. The immediate collapse and very poor condition of the patient combined with the fact that the uterus is not palpable abdominally should suggest it. However, in very fat patients, or in cases where the uterus is very soft and flabby, it is also not easy to feel it abdominally, and the careful doctor may often suspect the condition, when as a matter of fact the uterus is normal, and doing vaginal examinations to explore may do a great deal more harm than good. Sometimes, of course, the dimpling of the fundus can be felt: and very rarely, the uterus comes right outside the vulva with the placenta attached, as it did in the first case.

The diagnosis seems to rest on four points: first the patient is more shocked than the amount of bleeding would suggest, though cases are described in which this is not so; secondly, the difficulty in feeling the uterus; thirdly, although various methods of resuscitation are being used the response is poor; and lastly, the very unusual type of vaginal bleeding. In the first and third cases this was particularly noticeable. It is not the usual bright red hæmorrhage, but very dark red blood, which trickles away, and on supra-pubic pressure dark clots are expressed. Again it is not the same as the old black clots that are expressed after a concealed accidental hæmorrhage.

The treatment adopted was, first to deal with shock, by moderate warmth, raising the foot of the bed, morphia and blood transfusion ; when the patient has sufficiently recovered, as seen by the blood-pressure rising, it is probably best to replace the uterus under a general anæsthetic. The method that proved successful in all these cases was to place the patient on her back, or in lithotomy position, pass a catheter, and then with full sterile precautions introduce the whole hand into the vagina. The uterus is identified bulging down, but of course the cervix cannot be felt. It is important to make sure one is not dealing with a large fibroid protruding down into the vagina. (In that case of course the uterus should be felt above.) The fundus of the uterus is taken into the palm of the hand, and the rim where the uterus joins the vagina identified. The uterus is squeezed by the hand to reduce œdema, with pressure by the fingers in the fornices, and it is replaced gradually from below upwards, the fundus going back last. The thing *not* to do, is to try and replace the fundus first by pushing on it.

When the uterus is in the correct position ergometrine or pituitrin should be given. It is very useful in urgent cases to give it directly into the cervix with a long needle. Following this a hot intra-uterine douche should be administered. In giving the douche care must be taken not to raise the reservoir much above the level of the patient, as it must not be given under pressure, or fluid may be forced along the tubes into the peritoneal cavity. Normal saline at 118 deg. seems the best solution to use as it is completely non-irritating, even if some were to get into the veins of the placental site. Blood transfusion is of course absolutely life-saving in such cases, especially when the blood can be got in quickly, and continued during and after the operation.

The puerperium was, in every case, very satisfactory, and involution was complete.

I am grateful to the Medical Officer of Health, and to Dr. P. Phillips for allowing me to publish these cases.

